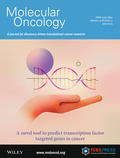# Issue Information

**DOI:** 10.1002/1878-0261.13237

**Published:** 2023-07-06

## Abstract

Basic cellular processes like cell division, replication, transcription and gene expression are often altered in cancer. The focus of this issue is on the molecular changes occurring in regulators of basic cellular processes that lead to malignancy.